# Antibody Production in Murine Polymicrobial Sepsis—Kinetics and Key Players

**DOI:** 10.3389/fimmu.2020.00828

**Published:** 2020-04-30

**Authors:** Oliver Nicolai, Christian Pötschke, Katrin Schmoeckel, Murthy N. Darisipudi, Julia van der Linde, Dina Raafat, Barbara M. Bröker

**Affiliations:** ^1^Immunology Department, Institute of Immunology and Transfusion Medicine, University Medicine Greifswald, Greifswald, Germany; ^2^Department of General Surgery, Visceral, Thoracic and Vascular Surgery, University Medicine Greifswald, Greifswald, Germany; ^3^Department of Microbiology and Immunology, Faculty of Pharmacy, Alexandria University, Alexandria, Egypt

**Keywords:** sepsis, splenectomy, T cell, antibody-secreting cells, IgM, IgG

## Abstract

Although antigen-specific priming of antibody responses is impaired during sepsis, there is nevertheless a strong increase in IgM and IgG serum concentrations. Using colon ascendens stent peritonitis (CASP), a mouse model of polymicrobial abdominal sepsis, we observed substantial increases in IgM as well as IgG of all subclasses, starting at day 3 and peaking 2 weeks after sepsis induction. The dominant source of antibody-secreting cells was by far the spleen, with a minor contribution of the mesenteric lymph nodes. Remarkably, sepsis induction in splenectomized mice did not change the dynamics of the serum IgM/IgG reaction, indicating that the marginal zone B cells, which almost exclusively reside in the spleen, are dispensable in such a setting. Hence, in systemic bacterial infection, the function of the spleen as dominant niche of antibody-producing cells can be compensated by extra-splenic B cell populations as well as other lymphoid organs. Depletion of CD4+ T cells did not affect the IgM response, while it impaired IgG generation of all subclasses with the exception of IgG3. Taken together, our data demonstrate that the robust class-switched antibody response in sepsis encompasses both T cell-dependent and -independent components.

## Introduction

Sepsis is still associated with astoundingly high morbidity and mortality despite improvements in intensive care (1–5). A systemic hyper-inflammatory phase (systemic inflammatory response syndrome, SIRS) is followed or accompanied by a compensatory anti-inflammatory response (compensatory anti-inflammatory response syndrome, CARS), with the risk of lethal (secondary) infections (6, 7). During the initial hyper-inflammatory phase, 40–50% of the T and B cell populations as well as innate immune cells go into apoptosis (8). Antigen presentation and T cell proliferation are impaired in the subsequent hypo-inflammatory phase, with a concomitant increase in concentrations of stress-induced anti-inflammatory glucocorticoids. These aforementioned effects, together with a Th2 cytokine bias, impair an effective immune response against primary or secondary infections (9–16). This explains the fact that mortality from sepsis mostly occurs during this later phase (17, 18).

It is well-documented that the antigen-specific B cell response in sepsis is strongly reduced (19–22). For example, Mohr et al. have shown an impaired primary B cell response against defined antigens (22). However, they have also observed an unspecific increase of serum IgM and IgG concentrations after cecal ligation and puncture, a commonly used mouse model of sepsis (22). However, details of the B cell response in sepsis that could explain that discrepancy are largely unknown (21, 23).

During an antigen-driven T cell-dependent (TD) immunoreaction against protein antigens, follicular B cells, which belong to the group of B-2 cells, are activated via the B cell receptor. With the help of activated T cells, they start to differentiate and form germinal centers, where class switch and somatic hypermutation take place. By the end of this process, affinity-matured plasma cells have developed that continuously secrete antibodies (24).

On the other hand, microbial components, which are systemically disseminated during sepsis, can activate B cells in a T cell-independent (TI) manner. For instance, TI-2 antigens (e.g., polysaccharides) crosslink B cell receptors and initiate a strong and long-lasting antigen-specific primary response (25). TI-1 antigens (e.g., lipopolysaccharide, LPS and bacterial DNA, CpG) activate B cells independent from the B cell receptor via toll-like receptors (TLRs), thereby inducing proliferation and antibody secretion (26, 27). In addition, TLR ligation itself can induce class switch recombination (28–31).

Though all naive and memory B cells in the mouse constitutively express TLRs (32–35), there are mainly two B cell subtypes, namely B-1 and marginal zone (MZ) B cells, which differentiate into antibody-secreting cells (ASC) soon after TLR-activation (34). Their antibody repertoire is restricted, polyreactive and lacking somatic hypermutation (36–38). These antibodies are produced to bridge the time gap until the adaptive response has sufficiently matured.

B-1 cells differ in their mode of activation, development, specificities and locations from follicular B cells. Their main reservoir are the pleural and peritoneal cavities, where they can be further subdivided based on their CD5 expression into B-1a (CD5+) and B-1b (CD5-) cells. In addition, they can be found in small proportions in all lymphoid organs and are prone to TI responses. They are selected during development based on a certain strength of self-binding. In strong contrast to follicular B cells, their BCR engagement does not lead to activation. They are able to switch to all IgG subclasses *in vitro*, whereas *in vivo* they produce natural antibodies mainly of the IgM, IgG3 and IgA isotype [reviewed extensively in (24, 38, 39)].

MZ B cells are located close to the marginal sinus in the murine spleen (40, 41), where they have direct access to blood-borne antigens (42, 43). Although they have the capacity to generate TD and TI responses (44–46), their main function is the TI response against blood pathogens. They differentiate very early into IgM- or IgG-secreting cells (43).

Follicular B cells (or B-2 cells) react only moderately or weakly to TI-1 antigens (34, 47, 48), but are classically the main producers of T cell-dependent, class-switched and hypermutated antibodies, which are produced in response to an antigen-specific TD response. They are found in peripheral lymphoid organs but also in the peritoneal cavity (PC) (49, 50).

In the present study, we set out to examine B cell reactions and antibody secretion in polymicrobial abdominal sepsis, with the aim of explaining disparities in research findings. For that purpose, we used two murine models for sepsis induction: (i) fecal-induced peritonitis (FIP): intraperitoneal (*i.p*.) injection of pooled cecal content of donor mice into recipient mice (51–53); (ii) colon ascendens stent peritonitis (CASP): continuous leakage of own gut content over a certain time, which mimics the clinical setting (54, 55). Whether splenic follicular or MZ B cells have a key role in the humoral response in sepsis was examined by explanting the spleen parallel to sepsis induction. In addition, CD4+ T cells were depleted before sepsis induction to determine the portion of the T cell-dependent and -independent humoral response.

## Materials and Methods

### Animal Experiments and Ethics Statement

Female C57BL/6 wild type (WT) mice were housed in a conventional, temperature-controlled animal facility (Central service and Research Institute for experimental animals of the University Medicine Greifswald) with a 12-h light and dark cycle, and provided with food and water *ad-libitum*. All animal experiments were performed in accordance with the German Animal Welfare Act (Deutsches Tierschutzgesetz) and the Federation of Laboratory Animal Science Associations (FELASA). The animal research protocol was approved by the animal ethics committee of the responsible local animal protection authority (LALLF, State Office for Agriculture, Food Safety and Fisheries Mecklenburg-Western Pomerania; numbers LALLF M-V/TSD/7221.3-1.1-052/07 and LALLF M-V/TSD/7221.3-1.2-013/09). All efforts were made to minimize animal suffering.

### Colon Ascendens Stent Peritonitis (CASP)

Colon ascendens stent peritonitis (CASP) surgery was performed as described before (54, 56). Briefly, mice were anesthetized with *i.p*. Ketamin (Ketanest, Parke-Davis GmbH, Berlin) and Xylazin (Rompun, Bayer Health Care, Leverkusen), 100/10 μg per g body weight, respectively. The abdomen was opened through a small incision and a 18G stent (Ohmeda AB, Helsingborg, Sweden) was implanted into their colon ascendens. After surgery, all animals were carefully monitored every 6 h (h) until the end of the experiment. Control animals received sham operations, without stent implantation. Animals were euthanized 10 or 14 days following CASP surgery.

### Fecal-Induced Peritonitis (FIP)

Sepsis was induced by introducing feces into the peritoneum using the method described by Wang et al. (57). In brief, littermates were anesthetized and euthanized. Fecal content (FC) was collected by cutting the *Ampulla ceci* and squeezing out the content. FC was homogenized in PBS to a final concentration of 100 mg/mL. The recipients received 7.25 × 10^5^ CFU *i.p*., whereas control animals were treated with PBS instead. At certain time points after sepsis induction (days 1, 3, 7, 14, 28, as well as at 12 weeks), animals were euthanized and the splenocytes isolated.

### Splenectomy

Following anesthesia, a midline laparotomy was performed. The cranial-dorsal and caudal-ventral spleen blood vessels were ligated with Mariderm 7/0, after applying yasergil-clips, and cut. The spleen was subsequently explanted.

### Antibody Assay

Mice were anesthetized, and blood was collected via the retrobulbar venous plexus using a microhematocrit capillary. Serum was collected after centrifugation of the coagulated blood at 16,000 × *g* for 10 min. Total serum IgM and IgG concentrations in murine serum were measured with the Milliplex® Mouse Immunoglobulin Isotyping Immunoassay (Millipore, MA, USA) according to the manufacturer's instructions. The samples were measured with the Luminex® 200 System (Bio-Rad Laboratories, Munich). Concentrations were calculated with the BioPlex Manager 5.0 software based on a provided standard.

### Enzyme Linked Immuno Spot Assay (ELISpot)

On assigned days, mice were euthanized under deep anesthesia and then spleen, mesenteric lymph nodes (MLN), femur and omentum were harvested for the preparation of single-cell suspensions. For spleen and MLN, 70 μm cell strainers (Sigma-Aldrich) were used. Bone marrow cells were prepared by flushing the femur with 10 mL cold PBS containing 5% fetal bovine serum (5% FBS/PBS). Cells were washed with cold 5% FBS/PBS (250 × *g*, 6 min, 4°C), and erythrocytes were lysed with sterile filtered ammonium chloride-buffer followed by another washing step.

A single-cell suspension of omentum was prepared by collagenase and DNAseI digestion (Roche Diagnostics GmbH, Mannheim, Germany). Briefly, the omentum was washed with PBS containing 5 mM EDTA for 1 min to get rid of the attached cells, followed by washing in HBSS containing 10% FBS and 0.01 M HEPES. The omentum was then cut into small pieces with a sterile scissor and incubated in 500 μL digestion buffer (PBS, 10% FBS, 0.01 M HEPES, 1.5 mg/mL collagenase D, 2 mg/mL DNAseI) for 30 min at 37°C with constant shaking (500 rpm). The resulting tissue was then mashed through a 70 μm cell strainer and washed twice in HBSS containing 10% FBS and 0.01 M HEPES (500 × *g*, 5 min, 4°C). The last step was then repeated with a 30 μm cell strainer.

All cells were resuspended in cold culture media (RPMI1640 supplemented with 50 μM 2-mercaptoethanol, 100 U/mL penicillin/streptomycin, 2 mM glutamine, 1 mM sodium pyruvate, 0.2% D-glucose, and 1% non-essential amino acids). The numbers of DAPI-negative and CD45-positive cells were determined as described in the flow cytometry section. The numbers of IgM- and IgG-secreting cells were determined using a mouse IgM and IgG ELISpotPLUS kit (Mabtech AB, Nacka Strand, Sweden). ELISpot was performed according to the manufacturer's instructions for *in vivo* activated cells (no additional activation required). Cells, titrated to 5,000–50,000 per well, were seeded in triplicates and incubated at 37°C for 16 h.

Spots were imaged using an ELISPOT plate reader (ImmunoSpot S5 Versa, Cellular Technology Limited) and counted using the Immunospot 5.0.3 Professional software (Cellular Technology Limited).

The number of ASCs per organ was calculated as follows:

cells per organ (BM only one femur) / cell number seeded × number of spots counted.

### Flow Cytometry

B cells were characterized using specific antibodies listed in Table 1, together with the necessary isotype controls (Table 2). B cell subpopulations were phenotypically defined according to the criteria listed in Table 3. Spleen cell suspensions were obtained as described before (62). Cell numbers were determined using BD TruCOUNT™ beads. One million cells were incubated with 2 μL Fc-Block for 15 min at 4°C. Then, 50 μL of the appropriate antibody-cocktail was added and incubated for further 30 min at 4°C. After washing (300 × *g*, 6 min) with FACS-buffer (BD FACSFlow Sheath Fluid, 2% FBS, 0.02% sodium azide), the pellet was resuspended in FACS-buffer and analyzed on a BD LSRII flow cytometer.

**Table 1:** Antibodies used for B cell characterization.

**Specificity**	**Fluorochrome**	**Isotype**	**Clone**	**Provider**	**Final conc. [μg/mL]**
B220	APC-A780	Rat IgG2a, κ	RA3-6B2	eBioscience	1
CD21	FITC	Rat IgG2b, κ	7G6	BD	10
CD23	PE	Rat IgG2a	B3B4	eBioscience	2
CD69	APC	Hamster IgG1	H1.2F3	BD	2
CD73	PE	Rat IgG2a, κ	TY/23	BD	4
CD95	PE-Cy7	Hamster IgG2	Jo2	BD	2
GL7	Alexa647	Rat IgM, κ	GL7	eBioscience	2
IgD	Horizon V450	Rat IgG2a, κ	11.26c2a	BD	5
IgM	PE-Cy7	Rat IgG2a, κ	R6-60.2	BD	10
CD45	FITC	Rat IgG2b, κ	30-F11	BioLegend	10

**Table 2:** Isotype controls.

**Isotype**	**Fluorochrome**	**Clone**	**Provider**	**Final conc. [μg/mL]**
Hamster IgG	APC	eBio299Arm	eBioscience	2
Hamster IgG2	PE-Cy7	B81-3	BD	2
Rat IgM, κ	Alexa647	RTK2118	BioLegend	5
Rat IgG2b	FITC		eBioscience	5
Rat IgG2a, κ	PE	R35-95	BD	4-8

**Table 3:** Definition of B cell populations.

**Population**	**Marker**	**References**	**Gating**
Follicular B cells	B220+ IgM+ IgD+ CD21int CD23+	(58, 59)	Supplementary Figure 1A
Marginal zone B cells	B220+ IgMhi IgDlo CD21+++ CD23-	(60)	Supplementary Figure 1A
Germinal center B cells	B220+ GL7+ CD95hi CD73int	(61)	Supplementary Figure 1B

### Depletion of CD4+ T Cells

For the depletion of CD4+ T cells, 150 μg rat anti-mouse CD4 mAb (GK1.5, in-house) was injected *i.p*. 1 and 3 days before CASP surgery. This efficiently depleted CD4+ cells without affecting CD8+ T lymphocytes (Supplementary Figure 2) as shown by FACS analysis using antibodies listed in Supplementary Table 3. Control mice received PBS instead. 14 days after depletion, the CD4 population in depleted, non-septic control mice had recovered to 40% compared with the non-depleted controls (own unpublished data).

### Statistical Analysis

Statistical analyses were performed using GraphPad Prism 6 for Windows (GraphPad Software, CA, USA). Data were assessed for significant differences using One-Way ANOVA with Bonferroni correction (Bonferroni *post-hoc* test) for selected pairs. *P* < 0.05 were considered significant.

## Results

### Strong Increase in Serum Immunoglobulin Concentrations After Sepsis

**Figure 1:**
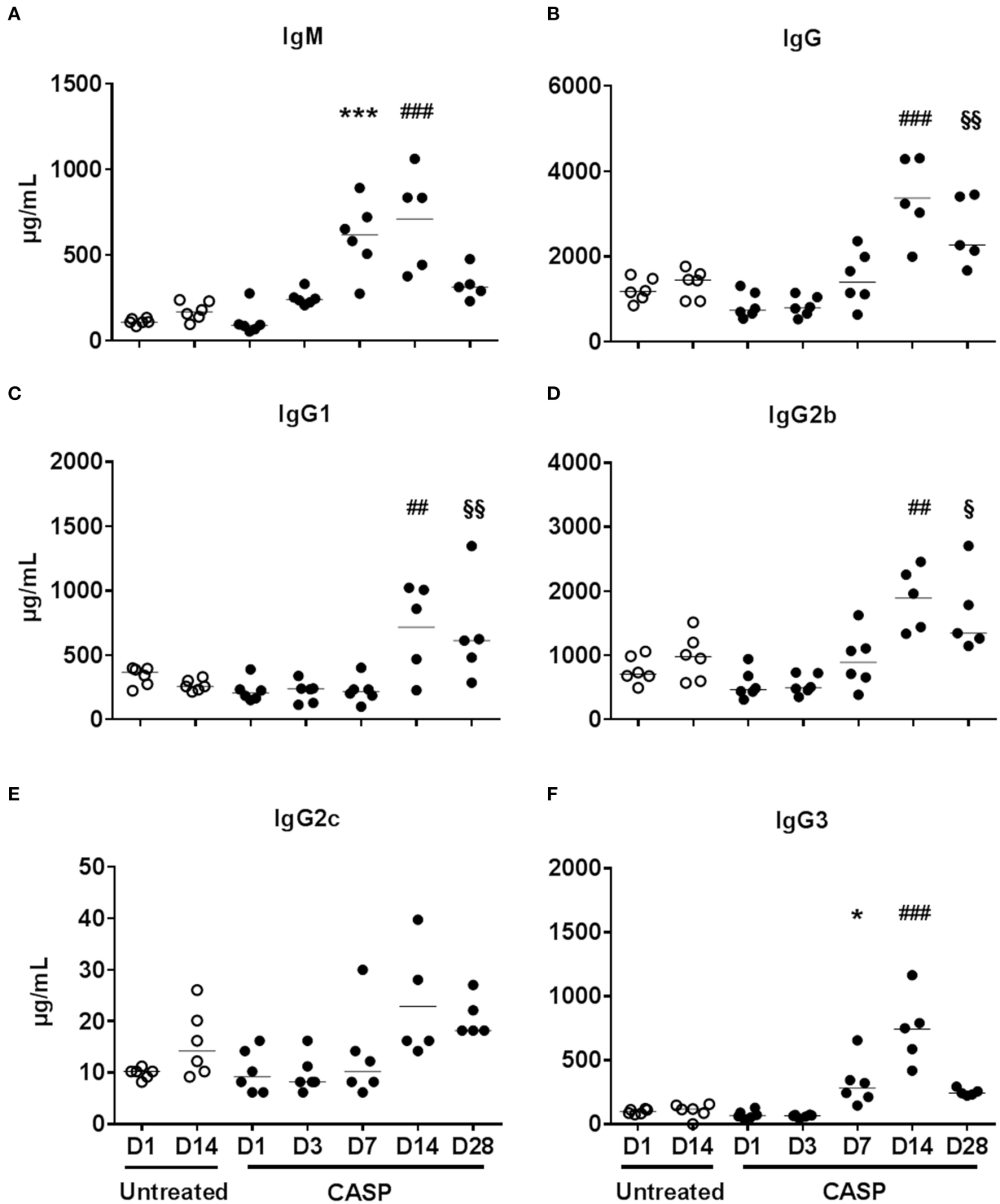
Serum immunoglobulin concentrations during the course of sepsis. Sepsis was induced by CASP-operation in female C57BL/6 mice. Untreated animals served as controls. At the indicated time points animals were anesthetized and blood was collected. Serum IgM **(A)** and IgG **(B)** concentrations, as well as the concentrations of all IgG-subtypes (C-F), were measured by Luminex®-technology. One of two similar experiments is shown here. We used the One Way ANOVA and Bonferroni *post hoc* test for selected pairs for statistical evaluation, and the mean is depicted in this figure. Significances are shown as number of symbols. one symbol *p* < 0.05; two symbols *p* < 0.01; three symbols *p* < 0.001. (*) CASP D7 vs. untreated D1, (^#^) CASP D14 vs. untreated D14, (^*$*^) CASP D28 vs. untreated D14; N = 5-6/group. The 95% confidence intervals of the differences of means are given in Supplementary Tables 1, 2.

During the course of sepsis, serum immunoglobulin (Ig) concentrations increased, reflecting B cell-activation and differentiation (Figure 1). In CASP, the IgM-serum concentration increased from 111.5 ± 17.71 μg/mL (CI 95%: 92.9–130; untreated d1) to 710.2 ± 291.1 μg/mL (CI 95%: 349–1,072) 14 days later (Figure 1A). At the same time the IgG-serum concentrations peaked at 3,372 ± 966.8 μg/mL (CI 95%: 2,171–4,572) at 14 days, compared to levels at day 1 [untreated day 1: 1,216 ± 270.6 μg/mL (CI 95%: 932–1,500)] (Figure 1B). This increase was distributed among all IgG-subtypes (Figures 1C–F), indicating at least partially T cell-dependent processes. These dynamics have also been observed in two other abdominal sepsis models (cecal ligation and puncture (CLP) and FIP, data not shown).

### The Spleen Is the Main Source of IgM- and IgG-Secreting Cells After Sepsis

**Figure 2:**
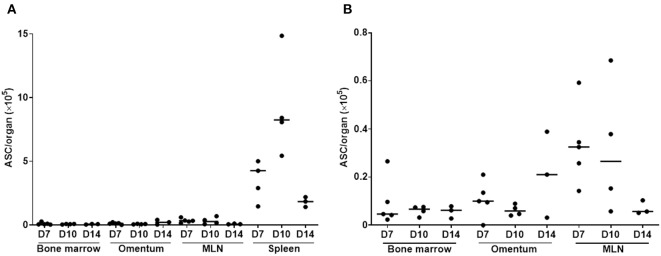
Antibody-secreting cells in lymphoid organs after sepsis. Sepsis was induced by CASP-operation in female C57BL/6 mice. At the indicated time points bone marrow, omentum, mesenteric lymph nodes (MLN) and spleen were harvested and cells isolated. Antibody-secreting cells (ASC, sum of IgM and IgG) per organ were measured with an ELISpot, and the median is depicted **(A)**. ASC/organ values below 1 × 10^5^ for bone marrow, omentum and MLN are separately shown **(B)**. *N* = 3–5.

Next, the source of the strong antibody reaction to sepsis was determined. Abdominal sepsis starts in the PC and is characterized by the systemic dissemination of pathogens and their products. Thus, both local and systemic immune responses are expected to take place. Locally, the parathymic lymph nodes are draining the PC (49, 63). They increase in size after sepsis induction but still have a much lower cell count compared to the spleen, ruling out a major contribution to the serum Ig response. Furthermore, the omentum and its lymph follicle-like structures, the so-called milky spots, have been ascribed a role in lymphocyte migration to and from the PC (49, 64, 65), while the mesenteric lymph nodes (MLN), an accumulation of relatively large lymph nodes in the PC, drain the gut and are probably not directly involved in the immune cell migration to or out of the PC. On the other hand, a systemic immune reaction will take place in the spleen due to the hematogenous spread of microbial compounds. Finally, the bone marrow might be involved as a source of immature as well as memory B cells, and a niche of long-lived plasma cells (66–68). ELISpot analyses clearly revealed the highest amount of ASCs in the spleen. At the peak of the response, namely 10 days after sepsis induction, around 10^6^ ASCs were counted in the spleens of septic animals. In addition, the MLNs seem to make a contribution to the antibody response, but the means differ by more than 20-fold (Figures 2A,B). In accordance with this, splenic follicular B cells, marginal zone B cells as well as germinal center B cells were rapidly activated in sepsis induced by FIP (within 24 h). The latter remained activated over a period of 12 weeks (Supplementary Figure 3).

### Spleen Cells, Including Marginal Zone B Cells, Are Not Necessary for the Production of Antibodies After Sepsis

**Figure 3:**
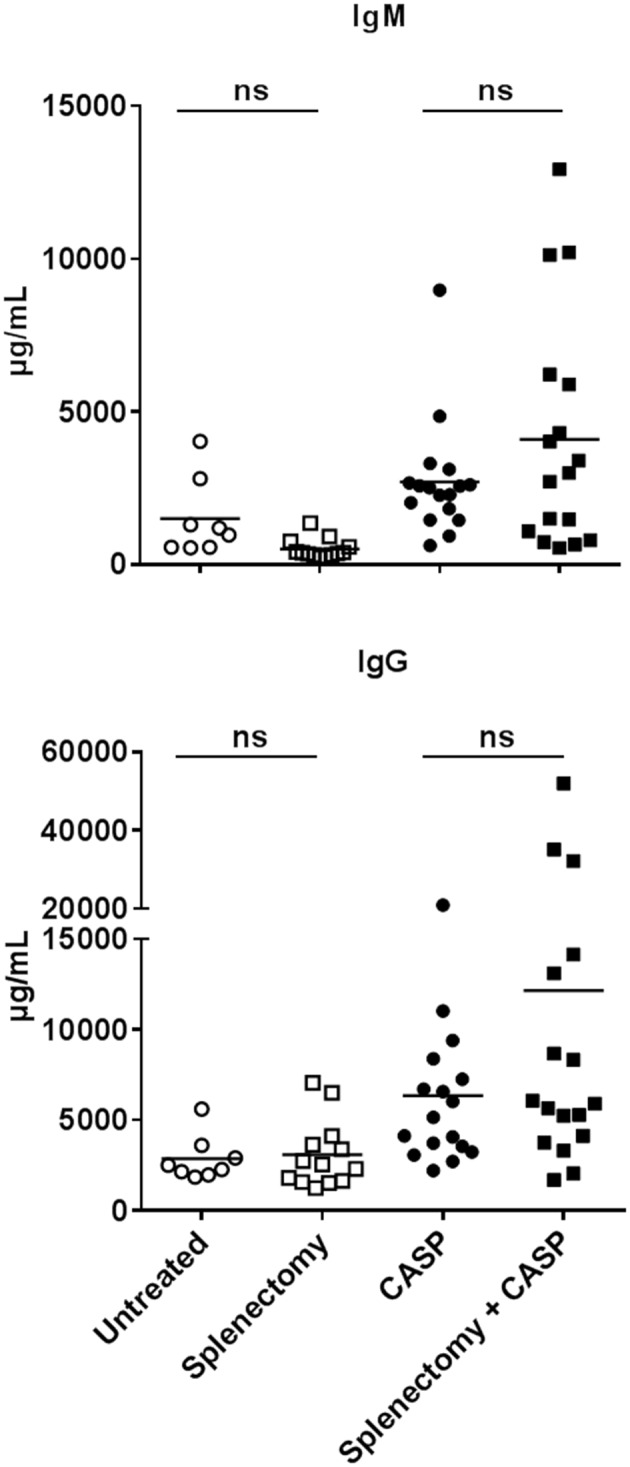
Serum IgM and IgG concentration 14 days after sepsis and splenectomy. Female C57BL/6 mice were CASP-operated and their spleen was explanted in parallel. Untreated, splenectomized-only and CASP-only animals served as controls. 14 days later animals were anesthetized and blood was collected. IgM and IgG serum concentrations were measured by Luminex®-technology. Shown is the mean of the collective data from two independent experiments with a similar tendency. We used the One Way ANOVA and Bonferroni *post-hoc* test for selected pairs for statistical evaluation. *p < 0.05; *N* = 8–17 per group.

Although we detected B cell activation and germinal center formation in the spleen, together with the majority of ASCs, it turned out that this organ was superfluous with regard to the observed strong increase of immunoglobulins after sepsis. To determine the input of splenic B cells to the overall humoral response, we splenectomized mice in parallel to CASP induction. Fourteen days later, there were no major changes in IgM or IgG serum concentrations as compared to the animals that received only CASP (Figure 3). Moreover, the lack of spleen had no effect on the induced IgG subclasses in the septic immune response (Supplementary Figure 4). The ostensible IgG-increase following splenectomy and CASP compared to CASP-only is due to three animals whose IgG2b concentrations increased strongly (Figure 3 and Supplementary Figure 4).

### The Antibody-Response After Sepsis Is Partially T Cell-Dependent

**Figure 4:**
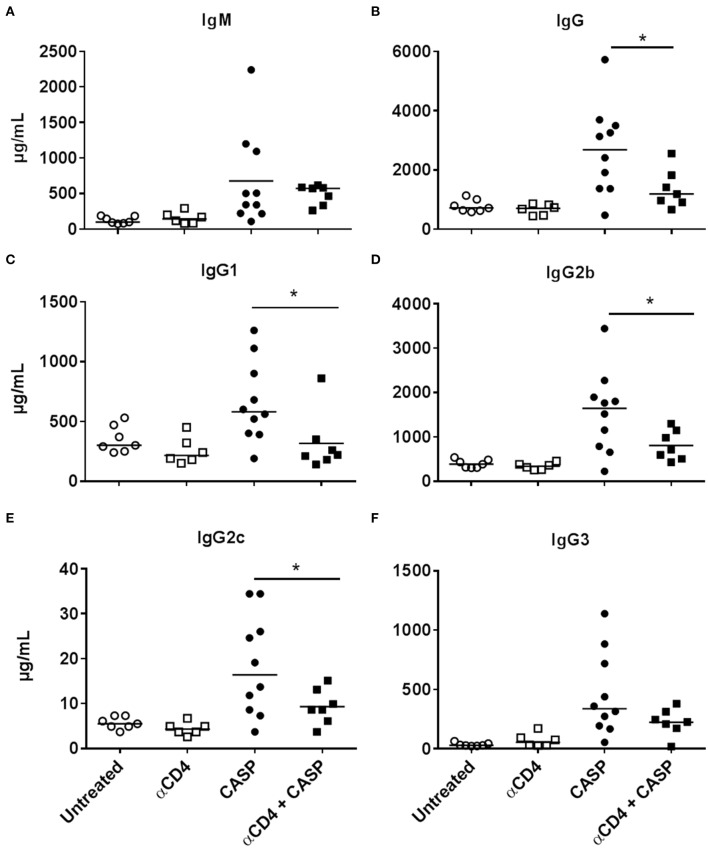
Serum Ig concentration 14 days after CASP and CD4+ cell depletion. Female C57BL/6 mice received 150 μg of a depleting anti-CD4 antibody (αCD4; Gk1.5) *i.p*. three and one day before sepsis induction via CASP. Control animals remained untreated or received the depleting antibody only. Fourteen days after CASP, animals were anesthetized and blood was collected. IgM **(A)** and IgG **(B)** serum concentrations, as well as IgG subclass concentrations **(C–F)**, were measured by Luminex®-technology. Shown is the mean of the collective data from two independent experiments with a similar tendency. We used the One Way ANOVA and Bonferroni *post-hoc* test for selected pairs for statistical evaluation *p < 0.05. *N* = 6–10 per group.

The generation of germinal centers as well as the strong increase in serum concentrations of all IgG subclasses makes a case for an antigen-driven TD Ig response in sepsis. Depleting CD4+ cells with an antibody (Gk1.5) prior to sepsis induction (Supplementary Figure 2) had no influence on IgM secretion (Figure 4A), but led to reduced serum IgG concentrations 14 days after CASP (Figure 4B). This supports the notion of a TD component in the B cell response. Interestingly, the decrease in serum IgG concentrations was absolute for IgG1 (returning to background levels), intermediate for IgG2b and IgG2c, but only in tendency for IgG3 (Figures 4C–F). Therefore, class switch in sepsis is evidently not exclusively dependent on T cells, but additionally driven by T cell-independent processes/antigens.

## Discussion

While battling invading pathogens, the systemic immune response causes collateral damage to the host, impairing life-securing homeostasis. Compensatory anti-inflammatory mechanisms and the necessary apoptotic loss of immune effector cells lead to immunosuppression, culminating in immunoparalysis (7, 8, 10, 12, 69, 70). It was hence assumed that the antibody response would also be impaired (70, 71). We have shown that serum IgM as well as IgG concentrations start to increase three to seven days after sepsis induction. The IgG increase was distributed among all IgG-subclasses, with the strongest relative increase observed for IgG3. IgG1, IgG2b, and IgG3 reached similar absolute serum concentrations of 1-2 mg/mL. The robust increase in serum Ig appears to be a general phenomenon in sepsis. Mohr et al. observed a similar increase 10 days after CLP (22), and our group obtained analogous results in a mouse model of FIP (data not shown). Brunner et al. have detected increased serum IgG concentrations in septic patients as early as 48 h after diagnosis (72).

An important role has been attributed to the spleen in the early defense against bacterial dissemination in the blood. Phagocytosis, endotoxin detoxification and antibody production are the main effector mechanisms. Especially MZ B cells carry TI responses (43, 73–75) and were deemed essential for successful pathogen eradication (42, 76). In accordance with this, the spleen was the main source of ASCs in our sepsis model, with a minor contribution of the MLNs. Moreover, we have shown an early activation of follicular and MZ B cells in the spleen, corroborating the results of other research groups (77). In accordance with what was observed by Kelly-Scumpia et al. (77), germinal centers were formed after 3 days. Four days later, high numbers of IgM and IgG-secreting cells were detected, especially in the spleen.

However, splenectomy did not impair the humoral immune response, as measured by the increase in serum antibody concentration. It seemed that splenic follicular as well as MZ B cells, despite being rapidly activated, were redundant. This was surprising in light of previous reports of a strongly reduced humoral response against bacterial antigens in splenectomized mice (78–81). In those studies, mice were infected 7–70 days following splenectomy. In yet another study, splenectomy led to a 75% reduction in B-1a cells after 6 days (60), which might explain the reduced humoral response in this setting. B-1a cells are known to participate in anti-bacterial and anti-viral responses (38, 82–84). We splenectomized the mice in parallel to CASP induction; hence extra-splenic B cell populations could still react to the multitude of antigens. Furthermore, in studies with *Borrelia hermsii*, MZ B cells did not play an important role either, because B-1 cells were the main producers of protective serum IgM (85, 86).

The fact that splenectomy did not decrease the serum IgM and IgG concentrations, despite the fact that we have clearly disclosed the spleen as the main source of ASCs, strongly argues for the hypothesis that most ASCs found in the spleen developed from immigrating B cells. These were probably peritoneal B cells, which – upon activation – left the peritoneum and migrated to spleen and peripheral lymph nodes (49, 86, 87). Some of these are obviously able to class switch to IgG. Our data also suggest that soon after splenectomy other lymphoid organs, be it mesenterial lymph nodes, the milky spots in the omentum and/or the parathymic lymph nodes, can compensate for the spleen.

Several groups have shown that antigen-specific priming is impaired after sepsis (21, 22, 88). So the question remains, what drives the strong antibody-increase in murine serum after sepsis? Obviously, microbial structures, such as LPS or CpG, flood the host system during sepsis, and are able to polyclonally activate B cells via their appropriate receptors (TLR4 or TLR9) (32, 89). Especially MZ B cells and B-1 cells differentiate into plasma cells (34, 35). Interestingly, LPS *per se* can induce class switch to IgG2b and IgG3, but also activated NKT cells, activated DCs and thrombocytes, all of which are abundant in sepsis, can at least partially compensate a lack of T cell help and promote TI antibody class switch (90–93).

On the other hand, the disseminating bacteria as well as dying host cells also confront the adaptive immune system with a wealth of antigens in the setting of sepsis. The pronounced germinal center reaction, in conjunction with the increase in all IgG subclasses support the idea that, besides polyclonal B cell activation, there may also be a significant antigen-driven component in the Ig response to sepsis.

Indeed, depletion of CD4+ T cells before sepsis reduced IgG-production, leaving the IgM-response intact. Especially IgG1 did not increase over the basal level in the absence of T cells. Although IgG2b, IgG2c, and IgG3 production was also less than in T cell competent septic animals, there was still a measurable increase. That shows that the IgG response to sepsis comprises both TD and TI components. Nevertheless, the observed significant TD IgG response seems to contradict reports of impaired antigen priming following sepsis induction (19, 21, 22, 88, 94, 95). The contrasting findings can, however, be reconciled by the following observations made by our research group (88): at sepsis onset, the T cell response to a primary antigen stimulus was not only fully intact but even enhanced, presumably through the adjuvant effects of the abundant pathogen-associated molecular patterns (PAMPs) and danger-associated molecular patterns (DAMPs) (88, 96). However, later during the disease, the T cell response to antigen priming was reduced, with a nadir 7 days after sepsis onset, where T cells in severely affected animals did not react at all (19, 88).

Our data reveal a strong humoral immune response in animals who survived sepsis. It is composed of T cell-dependent as well as T cell-independent components, takes place mainly in the spleen and probably involves the activation of all B cell populations. The task would now be to determine the antigen specificity of this Ig response in sepsis. This is addressed in the companion paper by Nicolai and co-workers (Nicolai et al., under revision).

In summary, in the present study, the origin of the strong antibody increase in sepsis was investigated, which identified the spleen as the main source of ASCs. Explanting the spleen parallel to sepsis induction revealed that both splenic follicular and MZ B cells are redundant in the humoral response to sepsis. Moreover, depletion of CD4+ T cells prior to sepsis induction highlighted the fact that both T cell-dependent and T cell-independent components govern the IgG response to sepsis.

## Data Availability Statement

All datasets generated for this study are included in the article/Supplementary Material.

## Ethics Statement

The animal study was reviewed and approved by Animal ethics committee of the local animal protection authority (LALLF, State Office for Agriculture, Food Safety and Fisheries Mecklenburg-Western Pomerania).

## Author Contributions

Conceptualization and project design: ON, CP, KS, and BB. Methodology and performance of experiments: ON, CP, and JL. Data evaluation: ON, CP and BB. Interpretation of data: ON, CP, KS, MD, JL, DR, and BB. Writing–original draft preparation: ON, MD, DR, and BB. Writing–review and editing. All authors critically reviewed the manuscript.

## Conflict of Interest

The authors declare that the research was conducted in the absence of any commercial or financial relationships that could be construed as a potential conflict of interest.

## References

[1] Slade E Tamber PS Vincent JL. The surviving sepsis campaign: raising awareness to reduce mortality. Crit Care. (2003) 7:1–2. 10.1186/cc1876 12617727 PMC154124

[2] Singer M Deutschman CS Seymour CW Shankar-Hari M Annane D Bauer M The third international consensus definitions for sepsis and septic shock (sepsis-3). JAMA. (2016) 315:801–10. 10.1001/jama.2016.0287 26903338 PMC4968574

[3] Rudd KE Johnson SC Agesa KM Shackelford KA Tsoi D Kievlan DR. Global, regional, and national sepsis incidence and mortality, 1990–2017: analysis for the global burden of disease study. Lancet. (2020) 395:200–11. 10.1016/S0140-6736(19)32989-7 31954465 PMC6970225

[4] Angus DC van der Poll T Severe sepsis and septic shock. N Engl J Med. (2013) 369:840–51. 10.1056/NEJMra1208623 23984731

[5] La Suarez De Rica A Gilsanz F Maseda E Epidemiologic trends of sepsis in western countries. Ann Transl Med. (2016) 4:325 10.21037/atm.2016.08.59 27713883 PMC5050194

[6] Hotchkiss RS Monneret G Payen D. Sepsis-induced immunosuppression: from cellular dysfunctions to immunotherapy. Nat Rev Immunol. (2013) 13:862–74. 10.1038/nri3552 24232462 PMC4077177

[7] Yende S Kellum JA Talisa VB Peck Palmer OM Chang C-CH Filbin MR. Long-term host immune response trajectories among hospitalized patients with sepsis. JAMA Netw Open. (2019) 2:e198686. 10.1001/jamanetworkopen.2019.8686 31390038 PMC6686981

[8] Francois B Jeannet R Daix T Walton AH Shotwell MS Unsinger J. Interleukin-7 restores lymphocytes in septic shock: the IRIS-7 randomized clinical trial. JCI Insight. (2018) 3:e98960. 10.1172/jci.insight.98960 29515037 PMC5922293

[9] Grimminger F Mayer K Seeger W. [Is there a reliable immunotherapy in infection?]. Internist. (1997) 38:541–52. 10.1007/PL00002644 9264999

[10] Hotchkiss RS Tinsley KW Swanson PE Schmieg RE Jr Hui JJ Chang KC. Sepsis-induced apoptosis causes progressive profound depletion of B and CD4+ T lymphocytes in humans. J Immunol. (2001) 166:6952–63. 10.4049/jimmunol.166.11.6952 11359857

[11] Tinsley KW Grayson MH Swanson PE Drewry AM Chang KC Karl IE. Sepsis induces apoptosis and profound depletion of splenic interdigitating and follicular dendritic cells. J Immunol. (2003) 171:909–14. 10.4049/jimmunol.171.2.909 12847261

[12] Wesche DE Lomas-Neira JL Perl M Chung CS Ayala A. Leukocyte apoptosis and its significance in sepsis and shock. J Leukoc Biol. (2005) 78:325–37. 10.1189/jlb.0105017 15817707

[13] Flohe SB Agrawal H Schmitz D Gertz M Flohe S Schade FU. Dendritic cells during polymicrobial sepsis rapidly mature but fail to initiate a protective Th1-type immune response. J Leukoc Biol. (2006) 79:473–81. 10.1189/jlb.0705413 16365154

[14] Hotchkiss RS Coopersmith CM McDunn JE Ferguson TA. The sepsis seesaw: tilting toward immunosuppression. Nat Med. (2009) 15:496–7. 10.1038/nm0509-496 19424209 PMC3786779

[15] Cavassani KA Carson WF Moreira AP Wen H Schaller MA Ishii M. The post sepsis-induced expansion and enhanced function of regulatory T cells create an environment to potentiate tumor growth. Blood. (2010) 115:4403–11. 10.1182/blood-2009-09-241083 20130237 PMC2881495

[16] Muenzer JT Davis CG Chang K Schmidt RE Dunne WM Coopersmith CM. Characterization and modulation of the immunosuppressive phase of sepsis. Infect Immun. (2010) 78:1582–92. 10.1128/IAI.01213-09 20100863 PMC2849407

[17] Osuchowski MF Welch K Siddiqui J Remick DG. Circulating cytokine/inhibitor profiles reshape the understanding of the SIRS/CARS continuum in sepsis and predict mortality. J Immunol. (2006) 177:1967–74. 10.4049/jimmunol.177.3.1967 16849510

[18] Adib-Conquy M Cavaillon J-M. Compensatory anti-inflammatory response syndrome. Thromb Haemost. (2009) 101:36–47. 10.1160/TH08-07-0421 19132187

[19] Schmoeckel K Mrochen DM Hühn J Pötschke C Bröker BM. Polymicrobial sepsis and non-specific immunization induce adaptive immunosuppression to a similar degree. PLoS ONE. (2018) 13:e0192197. 10.1371/journal.pone.0192197 29415028 PMC5802895

[20] Pötschke C Kessler W Maier S Heidecke C-D Bröker BM. Experimental sepsis impairs humoral memory in mice. PLoS ONE. (2013) 8:e81752. 10.1371/journal.pone.0081752 24312349 PMC3842948

[21] Sjaastad FV Condotta SA Kotov JA Pape KA Dail C Danahy DB. Polymicrobial sepsis chronic immunoparalysis is defined by diminished Ag-specific T cell-dependent B cell responses. Front Immunol. (2018) 9:2532. 10.3389/fimmu.2018.02532 30429857 PMC6220049

[22] Mohr A Polz J Martin EM Griessl S Kammler A Potschke C. Sepsis leads to a reduced antigen-specific primary antibody response. Eur J Immunol. (2012) 42:341–52. 10.1002/eji.201141692 22105154

[23] Gustave C-A Gossez M Demaret J Rimmelé T Lepape A Malcus C. Septic shock shapes B cell response toward an exhausted-like/Immunoregulatory profile in patients. J Immunol. (2018) 200:2418–25. 10.4049/jimmunol.1700929 29459404

[24] Nutt SL Hodgkin PD Tarlinton DM Corcoran LM. The generation of antibody-secreting plasma cells. Nat Rev Immunol. (2015) 15:160–71. 10.1038/nri3795 25698678

[25] Garcia De Vinuesa C O'Leary P Sze DM Toellner KM MacLennan IC. T-independent type 2 antigens induce B cell proliferation in multiple splenic sites, but exponential growth is confined to extrafollicular foci. Eur J Immunol. (1999) 29:1314–23. 10.1002/(SICI)1521-4141(199904)29:04<1314::AID-IMMU1314>3.0.CO;2-4 10229099

[26] Coutinho A Gronowicz E Bullock WW Moller G. Mechanism of thymus-independent immunocyte triggering. Mitogenic activation of B cells results in specific immune responses. J Exp Med. (1974) 139:74–92. 10.1084/jem.139.1.74 4128449 PMC2139516

[27] Krieg AM Yi AK Matson S Waldschmidt TJ Bishop GA Teasdale R. CpG motifs in bacterial DNA trigger direct B-cell activation. Nature. (1995) 374:546–9. 10.1038/374546a0 7700380

[28] Lutzker S Rothman P Pollock R Coffman R Alt FW. Mitogen- and IL-4-regulated expression of germ-line Ig gamma 2b transcripts: evidence for directed heavy chain class switching. Cell. (1988) 53:177–84. 10.1016/0092-8674(88)90379-0 2834063

[29] Severinson E Fernandez C Stavnezer J. Induction of germ-line immunoglobulin heavy chain transcripts by mitogens and interleukins prior to switch recombination. Eur J Immunol. (1990) 20:1079–84. 10.1002/eji.1830200520 1972677

[30] Mandler R Finkelman FD Levine AD Snapper CM. IL-4 induction of IgE class switching by lipopolysaccharide-activated murine B cells occurs predominantly through sequential switching. J Immunol. (1993) 150:407–18. 8419474

[31] Schnare M Barton GM Holt AC Takeda K Akira S Medzhitov R. Toll-like receptors control activation of adaptive immune responses. Nat Immunol. (2001) 2:947–50. 10.1038/ni712 11547333

[32] Pasare C Medzhitov R. Control of B-cell responses by toll-like receptors. Nature. (2005) 438:364–8. 10.1038/nature04267 16292312

[33] Barr TA Brown S Ryan G Zhao J Gray D. TLR-mediated stimulation of APC: distinct cytokine responses of B cells and dendritic cells. Eur J Immunol. (2007) 37:3040–53. 10.1002/eji.200636483 17918201 PMC2699383

[34] Genestier L Taillardet M Mondiere P Gheit H Bella C Defrance T. TLR agonists selectively promote terminal plasma cell differentiation of B cell subsets specialized in thymus-independent responses. J Immunol. (2007) 178:7779–86. 10.4049/jimmunol.178.12.7779 17548615

[35] Meyer-Bahlburg A Rawlings DJ Differential impact of toll-like receptor signaling on distinct B cell subpopulations. Front Biosci. (2012) 17:1499–516. 10.2741/4000 PMC3601656 22201817

[36] Cerutti A Cols M Puga I. Marginal zone B cells: virtues of innate-like antibody-producing lymphocytes. Nat Rev Immunol. (2013) 13:118–32. 10.1038/nri3383 23348416 PMC3652659

[37] Panda S Ding JL. Natural antibodies bridge innate and adaptive immunity. J Immunol. (2015) 194:13–20. 10.4049/jimmunol.1400844 25527792

[38] Baumgarth N. The double life of a B-1 cell: self-reactivity selects for protective effector functions. Nat Rev Immunol. (2011) 11:34–46. 10.1038/nri2901 21151033

[39] Savage HP Baumgarth N. Characteristics of natural antibody-secreting cells. Ann N Y Acad Sci. (2015) 1362:132-42. 10.1111/nyas.12799 26104151 PMC4679694

[40] Mebius RE Kraal G. Structure and function of the spleen. Nat Rev Immunol. (2005) 5:606–16. 10.1038/nri1669 16056254

[41] Weill JC Weller S Reynaud CA. Human marginal zone B cells. Annu Rev Immunol. (2009) 27:267–85. 10.1146/annurev.immunol.021908.132607 19302041

[42] Martin F Kearney JF. B-cell subsets and the mature -preimmune repertoire. Marginal zone and B1 B cells as part of a natural immune memory. Immunol Rev. (2000) 175:70–9. 10.1111/j.1600-065X.2000.imr017515.x 10933592

[43] Martin F Oliver AM Kearney JF. Marginal zone and B1 B cells unite in the early response against T-independent blood-borne particulate antigens. Immunity. (2001) 14:617–29. 10.1016/S1074-7613(01)00129-7 11371363

[44] Sha WC Liou HC Tuomanen EI Baltimore D. Targeted disruption of the p50 subunit of NF-kappa B leads to multifocal defects in immune responses. Cell. (1995) 80:321–30. 10.1016/0092-8674(95)90415-8 7834752

[45] Cariappa A Liou HC Horwitz BH Pillai S. Nuclear factor kappa B is required for the development of marginal zone B lymphocytes. J Exp Med. (2000) 192:1175–82. 10.1084/jem.192.8.1175 11034607 PMC2195875

[46] MacLennan IC Toellner KM Cunningham AF Serre K Sze DM Zuniga E. Extrafollicular antibody responses. Immunol Rev. (2003) 194:8–18. 10.1034/j.1600-065X.2003.00058.x 12846803

[47] Garcia De Vinuesa C Gulbranson-Judge A Khan M O'Leary P Cascalho M Wabl M. Dendritic cells associated with plasmablast survival. Eur J Immunol. (1999) 29:3712–21. 10556827 10.1002/(SICI)1521-4141(199911)29:11<3712::AID-IMMU3712>3.0.CO;2-P

[48] Fairfax KA Corcoran LM Pridans C Huntington ND Kallies A Nutt SL. Different kinetics of blimp-1 induction in B cell subsets revealed by reporter gene. J Immunol. (2007) 178:4104–11. 10.4049/jimmunol.178.7.4104 17371965

[49] Berberich S Dahne S Schippers A Peters T Muller W Kremmer E. Differential molecular and anatomical basis for B cell migration into the peritoneal cavity and omental milky spots. J Immunol. (2008) 180:2196–203. 10.4049/jimmunol.180.4.2196 18250426

[50] Berberich S Förster R Pabst O. The peritoneal micromilieu commits B cells to home to body cavities and the small intestine. Blood. (2007) 109:4627–34. 10.1182/blood-2006-12-064345 17289810

[51] Nguyen H-H Tran B-T Muller W Jack RS. IL-10 acts as a developmental switch guiding monocyte differentiation to macrophages during a murine peritoneal infection. J Immunol. (2012) 189:3112–20. 10.4049/jimmunol.1200360 22869902

[52] Jacobs S Sobki S Morais C Tariq M. Effect of pentaglobin and piperacillin on survival in a rat model of faecal peritonitis: importance of intervention timings. Acta Anaesthesiol Scand. (2000) 44:88–95. 10.1034/j.1399-6576.2000.440116.x 10669278

[53] Shrum B Anantha RV Xu SX Donnelly M Haeryfar SM McCormick JK. A robust scoring system to evaluate sepsis severity in an animal model. BMC Res Notes. (2014) 7:233. 10.1186/1756-0500-7-233 24725742 PMC4022086

[54] Maier S Traeger T Entleutner M Westerholt A Kleist B Hüser N. Cecal ligation and puncture versus colon ascendens stent peritonitis: two distinct animal models for polymicrobial sepsis: two distinct animal models for polymicrobial sepsis. Shock. (2004) 21:505–11. 10.1097/01.shk.0000126906.52367.dd 15167678

[55] Traeger T Koerner P Kessler W Cziupka K Diedrich S Busemann A Colon ascendens stent peritonitis (CASP)–a standardized model for polymicrobial abdominal sepsis. J Vis Exp. (2010) 18:2299 10.3791/2299 PMC3159662 21206468

[56] Zantl N Uebe A Neumann B Wagner H Siewert JR Holzmann B. Essential role of gamma interferon in survival of colon ascendens stent peritonitis, a novel murine model of abdominal sepsis. Infect Immun. (1998) 66:2300–9. 10.1128/IAI.66.5.2300-2309.1998 9573121 PMC108195

[57] Wang Z Rui T Yang M Valiyeva F Kvietys PR. Alveolar macrophages from septic mice promote polymorphonuclear leukocyte transendothelial migration via an endothelial cell Src kinase/NADPH oxidase pathway. J Immunol. (2008) 181:8735–44. 10.4049/jimmunol.181.12.8735 19050294

[58] Allman D Pillai S. Peripheral B cell subsets. Curr Opin Immunol. (2008) 20:149–57. 10.1016/j.coi.2008.03.014 18434123 PMC2532490

[59] Madan R Demircik F Surianarayanan S Allen JL Divanovic S Trompette A. Nonredundant roles for B cell-derived IL-10 in immune counter-regulation. J Immunol. (2009) 183:2312–20. 10.4049/jimmunol.0900185 19620304 PMC2772089

[60] Wardemann H Boehm T Dear N Carsetti R. B-1a B cells that link the innate and adaptive immune responses are lacking in the absence of the spleen. J Exp Med. (2002) 195:771–80. 10.1084/jem.20011140 11901202 PMC2193734

[61] Dogan I Bertocci B Vilmont V Delbos F Megret J Storck S. Multiple layers of B cell memory with different effector functions. Nat Immunol. (2009) 10:1292–9. 10.1038/ni.1814 19855380

[62] Busse M Traeger T Potschke C Billing A Dummer A Friebe E. Detrimental role for CD4+ T lymphocytes in murine diffuse peritonitis due to inhibition of local bacterial elimination. Gut. (2008) 57:188–95. 10.1136/gut.2007.121616 17965062

[63] Terasawa M Nagata K Kobayashi Y. Neutrophils and monocytes transport tumor cell antigens from the peritoneal cavity to secondary lymphoid tissues. Biochem Biophys Res Commun. (2008) 377:589–94. 10.1016/j.bbrc.2008.10.011 18854170

[64] Rangel-Moreno J Moyron-Quiroz JE Carragher DM Kusser K Hartson L Moquin A. Omental milky spots develop in the absence of lymphoid tissue-inducer cells and support B and T cell responses to peritoneal antigens. Immunity. (2009) 30:731–43. 10.1016/j.immuni.2009.03.014 19427241 PMC2754314

[65] Moon H Lee JG Shin SH Kim TJ. LPS-induced migration of peritoneal B-1 cells is associated with upregulation of CXCR4 and increased migratory sensitivity to CXCL12. J Korean Med Sci. (2012) 27:27–35. 10.3346/jkms.2012.27.1.27 22219610 PMC3247770

[66] Manz RA Thiel A Radbruch A. Lifetime of plasma cells in the bone marrow. Nature. (1997) 388:133–4. 10.1038/40540 9217150

[67] Chu VT Beller A Nguyen TT Steinhauser G Berek C. The long-term survival of plasma cells. Scand J Immunol. (2011) 73:508–11. 10.1111/j.1365-3083.2011.02544.x 21352257

[68] Weinstein JS Delano MJ Xu Y Kelly-Scumpia KM Nacionales DC Li Y. Maintenance of anti-Sm/RNP autoantibody production by plasma cells residing in ectopic lymphoid tissue and bone marrow memory B cells. J Immunol. (2013) 190:3916–27. 10.4049/jimmunol.1201880 23509349 PMC3622197

[69] Cohen J. The immunopathogenesis of sepsis. Nature. (2002) 420:885–91. 10.1038/nature01326 12490963

[70] Hotchkiss RS Karl IE. The pathophysiology and treatment of sepsis. N Engl J Med. (2003) 348:138–50. 10.1056/NEJMra021333 12519925

[71] Hotchkiss RS Nicholson DW. Apoptosis and caspases regulate death and inflammation in sepsis. Nat Rev Immunol. (2006) 6:813–22. 10.1038/nri1943 17039247

[72] Brunner M Krenn C Roth G Moser B Dworschak M Jensen-Jarolim E. Increased levels of soluble ST2 protein and IgG1 production in patients with sepsis and trauma. Intensive Care Med. (2004) 30:1468–73. 10.1007/s00134-004-2184-x 14991091

[73] Amlot PL Hayes AE. Impaired human antibody response to the thymus-independent antigen, DNP-Ficoll, after splenectomy. Implications for post-splenectomy infections. Lancet. (1985) 1:1008–11. 10.1016/S0140-6736(85)91613-7 2859463

[74] Ochsenbein AF Pinschewer DD Odermatt B Ciurea A Hengartner H Zinkernagel RM. Correlation of T cell independence of antibody responses with antigen dose reaching secondary lymphoid organs: implications for splenectomized patients and vaccine design. J Immunol. (2000) 164:6296–302. 10.4049/jimmunol.164.12.6296 10843683

[75] Altamura M Caradonna L Amati L Pellegrino NM Urgesi G Miniello S. Splenectomy and sepsis: the role of the spleen in the immune-mediated bacterial clearance. Immunopharmacol Immunotoxicol. (2001) 23:153–61. 10.1081/IPH-100103856 11417844

[76] Martin F Kearney JF. Marginal-zone B cells. Nat Rev Immunol. (2002) 2:323–35. 10.1038/nri799 12033738

[77] Kelly-Scumpia KM Scumpia PO Weinstein JS Delano MJ Cuenca AG Nacionales DC. B cells enhance early innate immune responses during bacterial sepsis. J Exp Med. (2011) 208:1673–82. 10.1084/jem.20101715 21746813 PMC3149216

[78] Jones JM Amsbaugh DF Prescott B. Kinetics of the antibody response to type III pneumococcal polysaccharide. II. Factors influencing the serum antibody levels after immunization with an optimally immunogenic dose of antigen. J Immunol. (1976) 116:52–64. 1446

[79] Amlot PL Grennan D Humphrey JH. Splenic dependence of the antibody response to thymus-independent (TI-2) antigens. Eur J Immunol. (1985) 15:508–12. 10.1002/eji.1830150516 2581791

[80] Teixeira FM Fernandes BF Rezende AB Machado RR Alves CC Perobelli SM. *Staphylococcus aureus* infection after splenectomy and splenic autotransplantation in BALB/c mice. Clin Exp Immunol. (2008) 154:255–63. 10.1111/j.1365-2249.2008.03728.x 18782329 PMC2612725

[81] Fernandes BF Rezende AB Alves CC Teixeira FM Farias RE Ferreira AP. Splenic autotransplantation restores IL-17 production and antibody response to *Streptococcus pneumoniae* in splenectomized mice. Transpl Immunol. (2010) 22:195–7. 10.1016/j.trim.2009.12.002 20036332

[82] Ochsenbein AF Fehr T Lutz C Suter M Brombacher F Hengartner H. Control of early viral and bacterial distribution and disease by natural antibodies. Science. (1999) 286:2156–9. 10.1126/science.286.5447.2156 10591647

[83] Baumgarth N Herman OC Jager GC Brown LE Herzenberg LA Chen J. B-1 and B-2 cell-derived immunoglobulin M antibodies are nonredundant components of the protective response to influenza virus infection. J Exp Med. (2000) 192:271–80. 10.1084/jem.192.2.271 10899913 PMC2193249

[84] Hayakawa K Hardy RR. Development and function of B-1 cells. Curr Opin Immunol. (2000) 12:346–53. 10.1016/S0952-7915(00)00098-4 10858035

[85] Alugupalli KR Michelson AD Joris I Schwan TG Hodivala-Dilke K Hynes RO. Spirochete-platelet attachment and thrombocytopenia in murine relapsing fever borreliosis. Blood. (2003) 102:2843–50. 10.1182/blood-2003-02-0426 12855586

[86] Ha SA Tsuji M Suzuki K Meek B Yasuda N Kaisho T. Regulation of B1 cell migration by signals through toll-like receptors. J Exp Med. (2006) 203:2541–50. 10.1084/jem.20061041 17060475 PMC2118139

[87] Yang Y Tung JW Ghosn EE Herzenberg LA. Division and differentiation of natural antibody-producing cells in mouse spleen. Proc Natl Acad Sci USA. (2007) 104:4542–6. 10.1073/pnas.0700001104 17360560 PMC1838637

[88] Schmoeckel K Traffehn S Eger C Potschke C Broker BM. Full activation of CD4+ T cells early during sepsis requires specific antigen. Shock. (2015) 43:192–200. 10.1097/SHK.0000000000000267 25243429 PMC4306536

[89] Lanzavecchia A Bernasconi N Traggiai E Ruprecht CR Corti D Sallusto F. Understanding and making use of human memory B cells. Immunol Rev. (2006) 211:303–9. 10.1111/j.0105-2896.2006.00403.x 16824137 PMC7165660

[90] Gao N Dang T Yuan D. IFN-gamma-dependent and -independent initiation of switch recombination by NK cells. J Immunol. (2001) 167:2011–8. 10.4049/jimmunol.167.4.2011 11489983

[91] Litinskiy MB Nardelli B Hilbert DM He B Schaffer A Casali P. DCs induce CD40-independent immunoglobulin class switching through BLyS and APRIL. Nat Immunol. (2002) 3:822–9. 10.1038/ni829 12154359 PMC4621779

[92] Elzey BD Tian J Jensen RJ Swanson AK Lees JR Lentz SR. Platelet-mediated modulation of adaptive immunity. A communication link between innate and adaptive immune compartments. Immunity. (2003) 19:9–19. 10.1016/S1074-7613(03)00177-8 12871635

[93] Elzey BD Sprague DL Ratliff TL. The emerging role of platelets in adaptive immunity. Cell Immunol. (2005) 238:1–9. 10.1016/j.cellimm.2005.12.005 16442516

[94] Wang F Wang YY Li J You X Qiu XH Wang YN. Increased antigen presentation but impaired T cells priming after upregulation of interferon-beta induced by lipopolysaccharides is mediated by upregulation of B7H1 and GITRL. PLoS ONE. (2014) 9:e105636. 10.1371/journal.pone.0105636 25144375 PMC4140801

[95] Gurung P Rai D Condotta SA Babcock JC Badovinac VP Griffith TS. Immune unresponsiveness to secondary heterologous bacterial infection after sepsis induction is TRAIL dependent. J Immunol. (2011) 187:2148–54. 10.4049/jimmunol.1101180 21788440 PMC3159846

[96] Cinel I Opal SM. Molecular biology of inflammation and sepsis: a primer. Crit Care Med. (2009) 37:291–304. 10.1097/CCM.0b013e31819267fb 19050640

