# Supplementary material for: Antibody Production in Murine Polymicrobial Sepsis—Kinetics and Key Players
Source: Front Immunol. 2020 Apr 30;11:828. doi: 10.3389/fimmu.2020.00828 (PMC7205023; doi:10.3389/fimmu.2020.00828)
Supplement: Supplementary file 1 [file Data_Sheet_1.docx]

Supplementary Material

# Supplementary Tables and Figures

## Supplementary Tables

**Supplementary Table 1**. 95% confidence interval (CI) of the differences of means for serum IgM and IgG concentrations during the course of sepsis.

| **Groups** | **IgM** | |  | **IgG** | |
| --- | --- | --- | --- | --- | --- |
|  | **CI** | **Sign.** |  | **CI** | **Sign.** |
| CASP D1vs untreated D1 | -221 - 221 | ns |  | -508 - 1230 | ns |
| CASP D3 vs untreated D1 | -359 - 84,1 | ns |  | -482 - 1260 | ns |
| CASP D7 vs untreated D1 | -715 - -272 | *** |  | -1140 - 600 | ns |
| CASP D14 vs untreated D14 | -769 - -305 | ### |  | -2920 - -1100 | ### |
| CASP D28 vs untreated D14 | -387 - 77,7 | ns |  | -2140 - -318 | §§ |

Abbreviations: ns, not significant; Sign., significance; vs, versus; two symbols: p <0.01; three symbols: p <0.001

**Supplementary Table 2:** 95% confidence interval (CI) of the differences of means for serum IgG subclass concentrations during the course of sepsis

| **Groups** | **IgG1** | | **IgG2c** | | **IgG2b** | | **IgG3** | |
| --- | --- | --- | --- | --- | --- | --- | --- | --- |
|  | **CI** | **Sign.** | **CI** | **Sign.** | **CI** | **Sign.** | **CI** | **Sign.** |
| CASP D1vs untreated D1 | -201 - 426 | ns | -10,2 - 9,53 | ns | -378 - 825 | ns | -169 - 218 | ns |
| CASP D3 vs untreated D1 | -193 - 435 | ns | -9,67 - 10,0 | ns | -371 - 832 | ns | -159 - 229 | ns |
| CASP D7 vs untreated D1 | -202 - 425 | ns | -13,1 - 6,57 | ns | -756 - 447 | ns | -416 - -28,3 | * |
| CASP D14 vs untreated D14 | -781 - -122 | ## | -17,5 - 3,14 | ns | -1550 - -286 | ## | -841 - -435 | ### |
| CASP D28 vs untreated D14 | -734 - -75,4 | §§ | -15,4 - 5,27 | ns | -1310 - -43,0 | § | -348 - 58,1 | ns |

Abbreviations: D, day; ns, not significant; Sign., significance; one symbol: p<0,05; two symbols: p <0.01; three symbols: p <0.001

**Supplementary Table 3: Antibodies for T cell staining**

| **Specificity** | **Conjugate** | **Isotyp** | **Clone** | **Company** |
| --- | --- | --- | --- | --- |
| CD3 | AlexaFluor® 647 | IgG2b, κ | 17A2 | eBioscience |
| CD4 | PE | Rat IgG2a, k | RM4-5 | BD |
| CD8a | FITC | Rat IgG2a, k | 53-6.7 | BD |
| CD11b | HV450 | Rat IgG2b, k | M1/70 | BD |

## Supplementary Figures


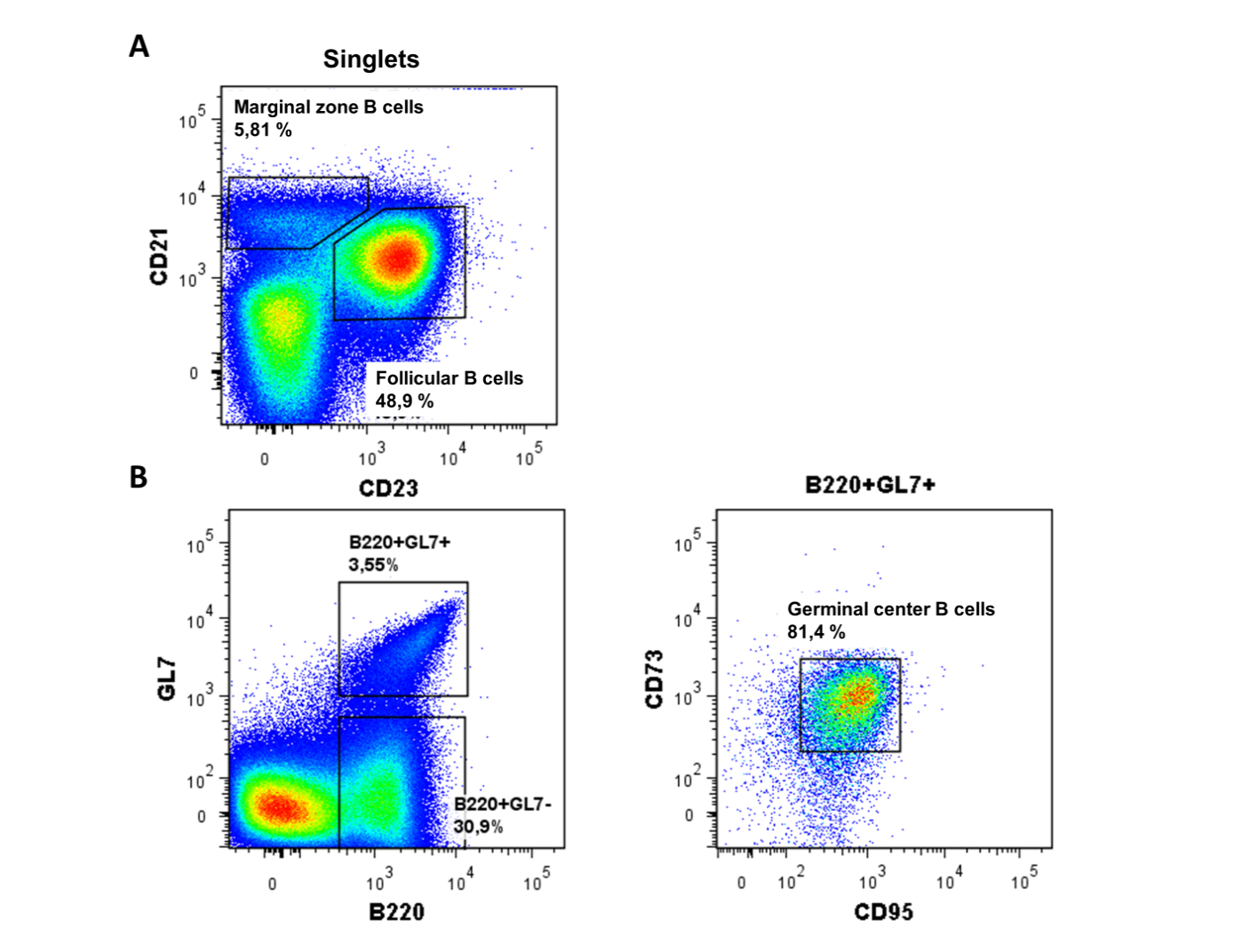


**Supplementary Figure 1**. Gating strategy for: (A) follicular and marginal zone B cells in spleen; (B) germinal center B cells in spleen and MLN (spleen is shown).


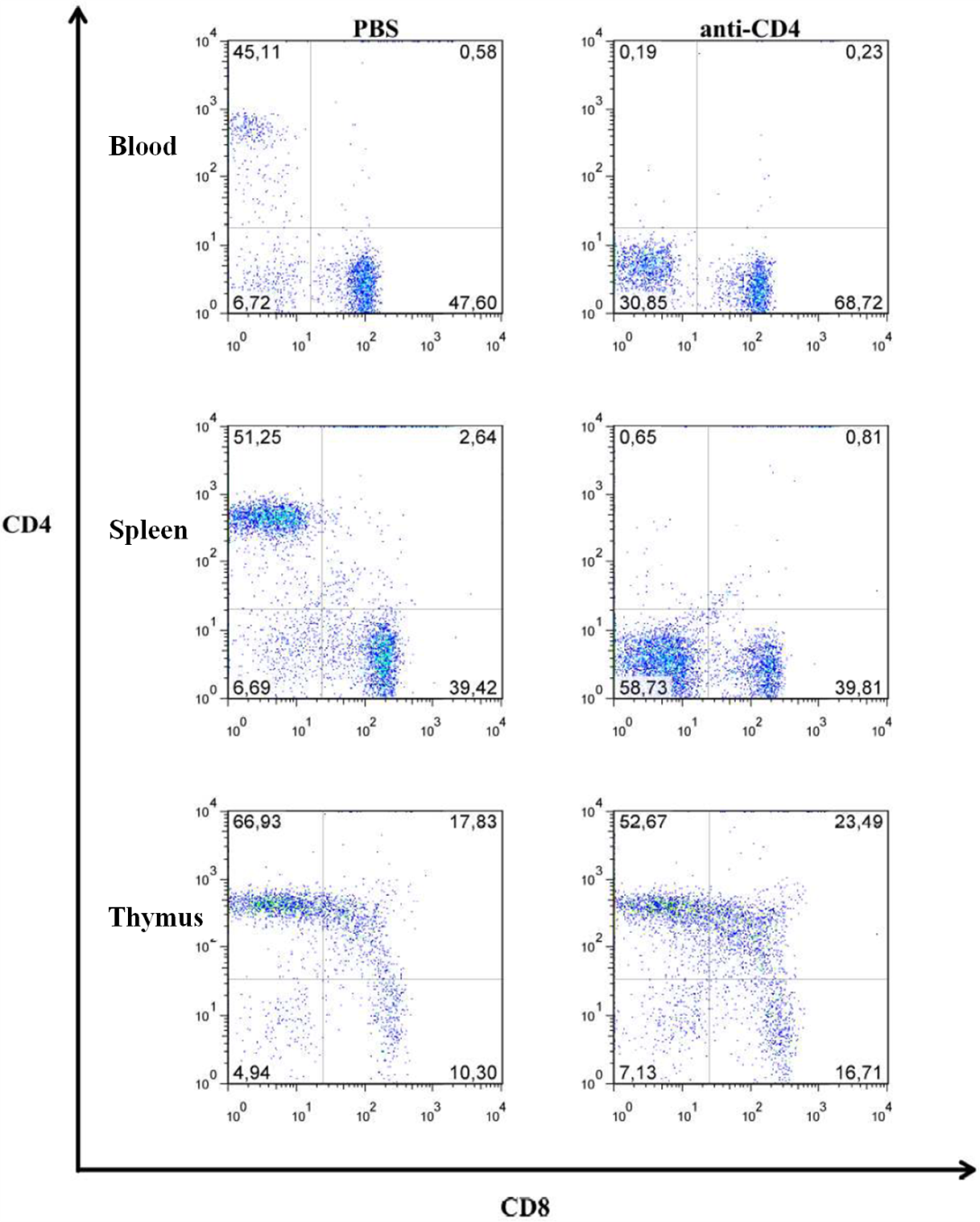


**Supplementary Figure 2. Verification of CD4+ cell depletion**

Female C57BL/6 mice received 150 µg of a depleting anti-CD4 antibody (αCD4; Gk1.5) *i.p*. 1 and 3 day before CASP surgery. Control animals received PBS instead. On day 0, animals were euthanized and the number of CD3+CD4+ T-cells and CD3+CD8+ T-cells in blood, spleen and thymus were determined. CD4+ T cells were successfully depleted in the periphery.

**Supplementary Figure 3: Early B cell activation after sepsis**

C57BL/6 mice received 7.25×10^5^ CFU-equivalents in PBS *i.p.* (FIP, fecal induced peritonitis). PBS-only treated animals served as controls. At the indicated time points, animals were euthanized and splenocytes isolated. CD69 expression on B220+IgM+IgD+CD21intCD23+ follicular (A) and B220+IgMhiIgDloCD21+++CD23- MZ (B) B cells (gMFI, geometric mean fluorescence intensity), as well as numbers of B220+GL7+CD95hiCD73int germinal center B cells (C) were measured by flow cytometry; the mean is depicted here. FACS staining revealed that splenic follicular and marginal zone (MZ) B cells became activated very early after sepsis, indicated by the expression of CD69 as early as 24 h after FIP (A and B) and B220+GL7+CD95hiCD73int germinal center B cells were formed, which persisted for at least 12 wk (C). We used the One Way ANOVA and Bonferroni post hoc test for selected pairs for statistical evaluation. N = 6-7 per group.

**Supplementary Figure 4. Serum IgG-subclass concentrations 14 days after sepsis and splenectomy**.

Female C57BL/6 mice were CASP-operated and their spleen was explanted in parallel. Untreated, splectomized-only and CASP-only animals served as controls. 14 days later animals were anesthetized and blood was collected. Serum concentrations of the IgG-subclasses were measured by the Luminex®-technology. Shown is the mean of the collective data from two independent experiments with similar tendency. We used the One Way ANOVA with Bonferroni correction (Bonferroni post hoc test) for selected pairs for statistical evaluation. N = 8-17 per group.
